# Nanotechnology for Early Cancer Detection

**DOI:** 10.3390/s100100428

**Published:** 2010-01-06

**Authors:** Young-Eun Choi, Ju-Won Kwak, Joon Won Park

**Affiliations:** Department of Chemistry, National Core Research Center for Systems Bio-Dynamics, Pohang University of Science and Technology, Pohang 790-784, Korea; E-Mails: luvangel@postech.ac.kr (Y.-E.C.); jwkwak@postech.ac.kr (J.-W.K.)

**Keywords:** cancer biomarkers, gold nanoparticles, quantum dots, carbon nanotubes, nanowires, microcantilevers

## Abstract

Vast numbers of studies and developments in the nanotechnology area have been conducted and many nanomaterials have been utilized to detect cancers at early stages. Nanomaterials have unique physical, optical and electrical properties that have proven to be very useful in sensing. Quantum dots, gold nanoparticles, magnetic nanoparticles, carbon nanotubes, gold nanowires and many other materials have been developed over the years, alongside the discovery of a wide range of biomarkers to lower the detection limit of cancer biomarkers. Proteins, antibody fragments, DNA fragments, and RNA fragments are the base of cancer biomarkers and have been used as targets in cancer detection and monitoring. It is highly anticipated that in the near future, we might be able to detect cancer at a very early stage, providing a much higher chance of treatment.

## 1. Introduction

Cancer diagnosis and treatment are of great interest due to the widespread occurrence of the diseases, high death rate, and recurrence after treatment. According to the National Vital Statistics Reports, from 2002 to 2006 the rate of incidence (per 100,000 persons) of cancer in White people was 470.6, in Black people 493.6, in Asians 311.1, and Hispanics 350.6, indicating that cancer is wide- spread among all races. Lung cancer, breast cancer and prostate cancer were the three leading causes of death in the US, claiming over 227,900 lives in 2007 alone, according to the National Cancer Institute. Cancer is also greatly feared due to recurrences, as although treatable, tumors can return after a period of time, even after chemotherapy, surgery, or radiotherapy.

Survival of a cancer patient depends heavily on early detection and thus developing technologies applicable for sensitive and specific methods to detect cancer is an inevitable task for cancer researchers. Existing cancer screening methods include: (1) the Papanicolau test for women to detect cervical cancer and mammography to detect breast cancer, (2) prostate-specific antigen (PSA) level detection in blood sample for men to detect prostate cancer, (3) occult blood detection for colon cancer, and (4) endoscopy, CT scans, X-ray, ultrasound imaging and MRI for various cancer detection. These traditional diagnostic methods however are not very powerful methods when it comes to cancer detection at very early stages. As well, some of the screening methods are quite costly and not available for many people. Therefore, the development of technology that is specific and reliable for detecting cancers at early stages and is easily accessible so that it can function as the first-line guidance is of utter importance.

Biomarkers and nanotechnology, two mainstream fields in development of powerful diagnostic methods, are being extensively studied these days. This review will cover developments in cancer diagnosis methods using biomarkers and nanotechnology with an emphasis on the studies focusing largely on nanomaterials conducted in the past three years.

## 2. Biomarkers

A biomarker is an indicator of a biological state of disease. It is characteristic of a specific state and therefore can be used as a marker for a target disease. These biomarkers can be used to study cellular processes, and monitor or recognize disruption or alterations in the cellular processes of cancer cells. These results could provide us with information on the underlying mechanism of the initiation of a disease, the process of aggravation, and ultimately provide a method to diagnose and treat the disease with appropriate measures at desired time.

A biomarker can be a protein, a fragment of a protein, DNA, or RNA-based. Biomarkers, specifically cancer biomarkers, are an indication of cancer and by detecting them the existence of that specific cancer can be verified. Alongside the development of proteomic technologies, many protein biomarkers have been discovered for many types of cancer. As well, with DNA methylation analysis researchers have also been able to discover DNA biomarkers for some of the widely spread cancers. Some of the biomarkers that are currently in use are listed below (Table 1).

Biomarkers in relation with nanotechnology and biosensors have opened up a new era of early cancer diagnosis and precise drug delivery. Therefore thorough reviews on biomarkers have been written due to their high importance [26–29].

## 3. Nanomaterials and Biomarkers

Nanotechnology has been developing rapidly during the past few years and with this, properties of nanomaterials are being extensively studied and many attempts are made to fabricate appropriate nanomaterials. Due to their unique optical, magnetic, mechanical, chemical and physical properties that are not shown at the bulk scale, nanomaterials have been used for more sensitive and precise biomarker detection. Nanomaterials that have been applied to sensing cancer biomarkers vary from gold nanoparticles, quantum dots, magnetic nanoparticles, carbon nanotubes and nanowires [30–32].

### 3.1. Gold Nanoparticles

Gold nanoparticles (GNPs) have been in the bio-imaging spotlight due to their special optical properties. GNPs with strong surface-plasmon-enhanced absorption and scattering have allowed them to emerge as powerful imaging labels and contrast agents. They have better absorption and scattering bands than conventional organic dyes, the cross section of the bands going up to four to five orders of magnitude higher [33]. Furthermore, GNPs have been proven to be more biocompatible, less cytotoxic, and resistant to photobleaching according to human cell experiments [34]. Another unique property of the GNPs is the size-tunable optical properties. According to their size and shape, GNPs can absorb and scatter light from the visible to near-infrared (NIR) region [35].

**Figure 1.**
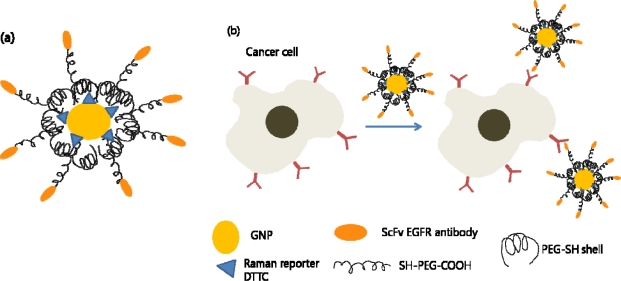
Cancer cell targeting and spectroscopic detection using antibody-conjugated SERS nanoparticles. (a) Modified gold nanoparticle with Raman reporter and targeting molecule. (b) Schematic illustration of the nanoparticles targeting the cancer cells.

GNPs have been extensively studied, especially in the medical area, and have been used as colorimetric biosensors [36–38], cancer imaging [39,40], cancer therapy [41–43], and drug delivery [44]. They have been found to amplify the efficiency of Raman scattering and thus have been proposed as a novel tag. A class of nontoxic nanoparticles for *in vivo* tumor targeting based on pegylated colloidal gold (colloidal gold coated with a protective layer of polyethylene glycol) and surface-enhanced Raman scattering (SERS) has been reported [45]. These gold nanoparticles have been encoded with Raman reporters and conjugated with ScFv antibody for *in vitro* and *in vivo* tumor targeting, recognizing the epidermal growth factor receptor (EGFR) which is a popular biomarker used in cancer targeting (Figure 1).

These SERS GNPs with ScFv antibody to target EGFR were more than 200 times brighter than NIR emitting quantum dots, and allowed spectroscopic detection of small tumors (0.03 cm^3^) at penetration depth of 1–2 cm.

**Figure 2.**
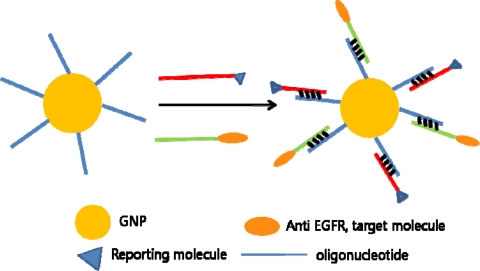
Functionalization of GNPs through hybridization oligonucleotides.

For GNPs as stable and versatile molecular imaging agents, a complementary oligonucleotide-based approach has been proposed. A 5′-thiol-modified and 3′-NH_2_-modified oligonucleotide was coated onto the nanoparticles and subsequently conjugated with anti-EGFR proteins through DNA-DNA hybridization. Through this study, gold nanoparticles have proven to be effective reflectance contrast agents for molecular imaging. As well, by hybridizing oligonucleotides with reporting molecule, the modified GNPs could also be used as a multifunctional contrast agent and be imaged with fluorescent confocal microscopy in parallel (Figure 2). These modified GNPs have been proven to be superior to contrast agents where the protein was adsorbed directly, in terms of size and stability [46].

Plasmon resonance coupling between closely spaced metal nanoparticles have been applied in *in vitro* bioanalytical assays. However a recent study has been conducted where plasmon resonance coupling was used for *in vivo* molecular imaging of carcinogenesis. Anti-EGFR antibodies were conjugated to gold nanoparticles and these nanoparticles were used to obtain information on the over-expression and nanoscale spatial relationship of EGFRs in cell membranes. EGFR-mediated aggregation of GNPs results in color shift and a contrast ratio much superior than those with fluorescent dyes when normal and precancerous epithelium were imaged *in vivo* [47].

**Figure 3.**
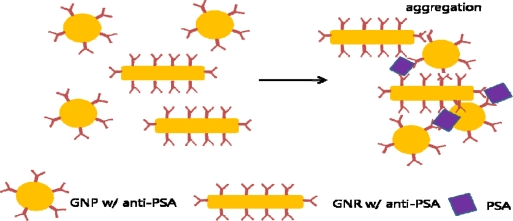
Aggregation of GNPs and gold nanorods in the presence of PSA, leading to DLS analysis for the immunoassay.

Dynamic light scattering (DLS) analysis can also be used for biomarker sensing. A combination of GNPs and gold nanorods conjugated with anti-Prostate Specific Antigen (PSA) antibody was used as a one-step homogeneous immunoassay for cancer biomarker detection. Through DLS analysis, the relative ratio of nanoparticle aggregate *versus* nonaggregated nanoparticles can be measured quantitatively. The relative ratio should therefore increase according to the amount of antigen in sample solution and this relationship is the basis of the homogeneous immunoassay (Figure 3). This study was conducted in solution, contrary to the traditional plate-based immunoassay, therefore allowing better mixing between antigen and antibody. This enables biomarker detection at very low concentrations [48].

**Figure 4.**
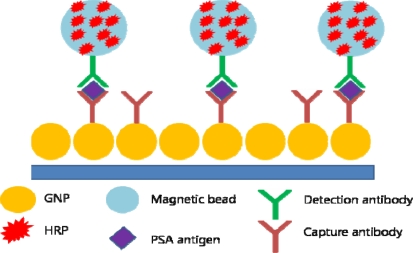
GNP electrode and magnetic beads functionalized with multiple enzyme labels.

Gold nanoparticles are used as molecular imaging agents, but GNP film electrodes have also been proven to be useful in detecting cancer biomarker proteins. By applying multilabeled detection antibody-magnetic bead bioconjugates, an ultrasensitive electrochemical immunosensor for cancer biomarker proteins has been designed. Magnetic beads have been conjugated with horse radish peroxide (HRP) and a secondary antibody, providing multiple enzyme labels for each PSA to be detected. On the surface of the GNP electrode, capture antibodies have been attached for PSA antigen binding. By applying voltage and H_2_O_2_, a sensitive immunosensor was realized (Figure 4). The authors claimed the immunosensor was better than a previously reported carbon nanotube (CNT) forest immunosensor with multiple labels on the CNTs [49].

In addition to spherical gold nanoparticles, gold nanoshells [50,51] and gold nanorods [52] have been applied to biomarker detection. They have been of particular interest because they can absorb and emit light at NIR region providing deep tissue penetration which is very important in *in vivo* imaging. As well, these nanoparticles have been very useful as a photothermal cancer treatment agent.

### 3.2. Quantum Dots

Quantum dots (QDs) are semiconducting, light-emitting nanocrystals that have emerged as a powerful molecular imaging agent since their discovery. QDs are an exciting material to work with due to their unique optical properties compared to traditional organic fluorescent labels [53]. Organic fluorescent dyes have several drawbacks that have limited their usefulness as molecular imaging tags. Their low photobleaching threshold and broad absorption/emission peak width have hindered their use in long term imaging and multiplexing (detecting multiple labels simultaneously) [54]. QDs have properties that overcome these limitations of the organic fluorescent dyes including high resistance to photobleaching [55], broad-band absorption with narrow emission bands ranging from UV to NIR [56,57], and size tunable emission bands [58,59]. These exceptional optical properties of QDs have made them an exciting field of study for many researchers in search of molecular imaging tools for better cancer diagnosis.

**Figure 5.**
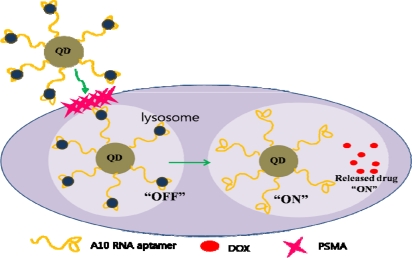
Schematic design of the multifunctional nanoparticle. QDs conjugated with an aptamer targets the cancer biomarker. By releasing the drug from the conjugate, both the QD and drug recovers its fluorescent property.

A multifunctional nanoparticle with biomolecules conjugated to QDs has been used in cancer targeting and drug delivery [60]. A10 RNA, aptamer that recognizes prostate specific membrane antigen (PSMA), was conjugated to the QD to target cancer cells [61]. Doxorubicin (DOX), a well known anthracycline drug with fluorescent properties, was intercalated in the conjugate. This conjugate offers an exciting method of imaging cancer cells. The intercalated DOX within A10 RNA conjugated to the QD quenches the fluorescence of both DOX and QD. When the QD-aptamer (DOX) conjugate finds the target cancer biomarker, it is taken into the cancerous cell through endocytosis. When DOX is released from the conjugate, both of them recover their fluorescent properties and thus can be imaged. With this design, the multifunctional nanoparticle can be fabricated so that cancer biomarkers can be detected precisely and the drug is delivered within the cancer cell, giving high specificity (Figure 5).

**Figure 6.**
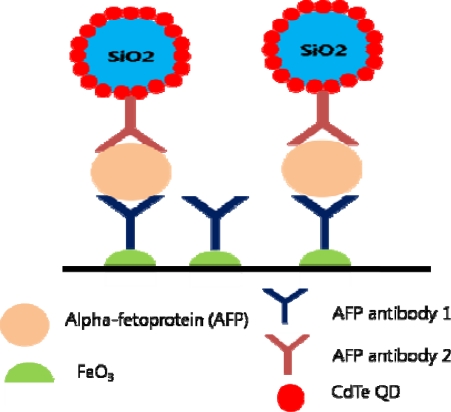
QD functionalized Si nanoparticles for signal amplification.

QDs can be used as signal amplifying agents in ultrasensitive cancer biomarker detection [62]. A recent study has been conducted with QD functionalized nanoparticles in immunoassays, targeting alpha-fetoproteins (AFPs) [63]. CdTe QDs have been coated on SiO_2_ particles and through anodic stripping voltammograms, the Si/QD/antibody showed increased oxidation current of Cd^2+^ proving its signal amplifying ability. Increased amount of QDs per biomarker make the detection more sensitive, thus enabling detection even at low concentration (Figure 6).

Magnetic particles have also been functionalized with QDs for cancer targeting, separation and imaging [64]. A high fluorescent multi-labeling could be achieved with this conjugation, providing both magnetic manipulation and multicolor fluorescent images. By immobilizing anti-epithelial cell adhesion molecule (EpCAM) antibody, this conjugate targeted tumor cells circulating tumor cells [65]. The conjugation of magnetic particle, QDs, and antibody is however very complex requiring multiple steps. Therefore many studies are conducted to find out easier methods to fabricate them.

QDs have also been integrated into nano-bio-chips (NBCs) for detecting multiple cancer biomarkers [66]. Recently, an exciting new technique has been developed using QDs in nano-bio-chips. QD-labeled antibodies were used for multiple-color-fluorescence transduction signaling NBCs in combination with antigen capture by a microporous agarose bead array held in microfluidics [67]. Cancer biomarkers of interest were carcinoembryonic antigen (CEA), cancer antigen 125 (CA125), and Her-2/*neu* in serum and saliva samples. This type of miniaturized chip proved to be superior to traditional enzyme-linked immunosorbent assay (ELISA), reducing the detection limit by nearly two orders of magnitude.

### 3.3. Other Nanoparticles

Other various nanoparticles have also been investigated for cancer biomarker detection. Metal nanoparticles, magnetic nanoparticles, silica nanoparticles and many other have caught the attention of researchers due to their unique properties.

Encapsulated phase change nanoparticles have been investigated as a multiplexed highly sensitive cancer biomarker detection agent [68]. Phase change nanoparticles have unique thermophysical properties. Phase change materials (PCM) can absorb thermal energy without temperature rise during the melting process. Heat transfer from surrounding environment to the core of PCM takes time in practice and that broadens the melting peak at temperature scan of a constant rate. By silica encapsulation, changes in composition of the metal within can be prevented, thus providing thermal barcodes with melting peaks broadening according to the concentration of the target molecule.

Silica nanoparticles doped with fluorescence resonance energy transfer (FRET) dyes have been investigated as simultaneous and multiplexed detection [69]. Recently, a study has been conducted using these FRET nanoparticles to monitor cancer cells [70]. By modifying the nanoparticles with aptamers targeting T-cell leukemia and B-cell lymphoma and by changing doping ratio of the dyes trapped inside the silica shell, a variety of fluorescent emission spectra could be obtained with a single excitation wavelength.

**Figure 7.**
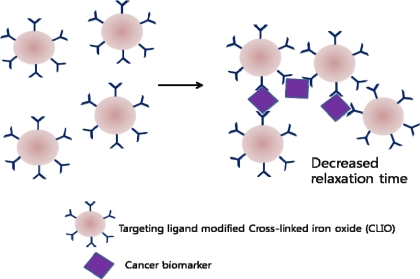
Mechanism of decrease in relaxation time due to magnetic nanoparticle aggregation.

Magnetic nanoparticles have been widely used in cancer cell imaging. By using the principle of decrease in transverse relaxation time due to aggregation of magnetic nanoparticles in presence of target molecules, concentration of cancer biomarkers could be measured [71]. This device allowed *in vivo*, local environment monitoring for cancer biomarkers and could be left implanted after tumor surgery. This exciting new method is expected to be a convenient method for continuous monitoring since biopsies do not have to be performed for each screening. A similar approached has also been used in the past to develop a biosensor to detect cancer biomarkers in turbid samples (blood, urine, and sputum) [72]. When magnetic particles aggregate through affinity ligands to the molecular target, a decrease in the bulk spin-spin relaxation time of surrounding water molecules occurs. This could be used as a chip-based nuclear magnetic resonance (NMR) system, and with miniaturization and multiplexing, detection of various biomarkers within a small sample volume could be achieved (Figure 7).

### 3.4. Carbon Nanotubes

After the discovery of fullerene, carbon nanotubes (CNTs) have been re-discovered in 1991 by Iijima [73]. Since then, CNTs have been constantly in the spotlight and have emerged as a powerful sensing vehicle due to their exciting properties. The conductance of the semiconducting CNT changes when biomolecules are adsorbed on the walls, causing changes in local electrostatic environment. Many exceptional properties of CNTs allow them to be applied for sensing biomarkers electrochemically; CNTs provide high surface-to-volume ratios, mediate fast electron-transfer and can be functionalized with almost any desired chemical species. It is also of great advantage to use carbon nanotubes because label-free detection of cancer biomarkers is possible. Label-free detection is based on molecular recognition events, requiring fewer steps in sample preparation compared to labeled-detection. Of course not only electrochemical biosensing is possible with CNTs, but also incorporating label molecules for imaging is also possible. Numerous studies on CNT-based biosensors have been conducted since the development of CNT electrodes for low potential detection of NADH [74–79].

**Figure 8.**
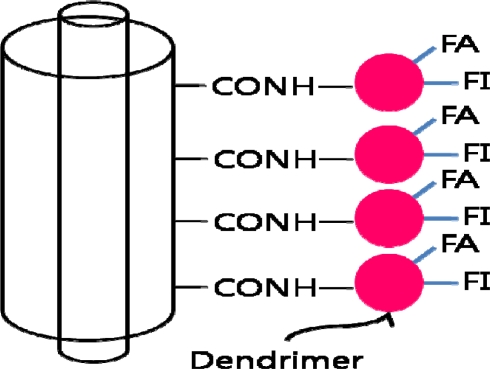
MWNT functionalized with fluorescein isothiocyanate (FI) and folic acid (FA) modified amine-terminated dendrimers. FA is for targeting cancer cells that over-expresses FA receptors and FI dye for imaging.

Current developments in CNT-based cancer detection focus on a more precise and sensitive detection of the cancer biomarkers. A multifunctional dendrimer-modified multiwalled carbon nanotube (MWNT) has been recently developed. The dendrimers, poly(amidoamine), are highly branched, monodispersed macromolecules with well-defined architecture making them an ideal platform to covalently link molecules of interest (Figure 8). This approach allows single-step modification of the CNTs for cancer cell targeting and imaging [80].

**Figure 9.**
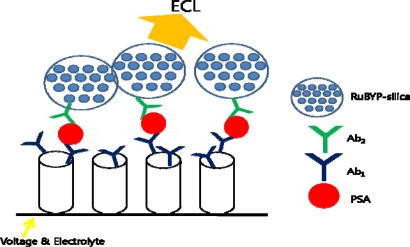
SWNT forest with ECL nanoparticles as sandwich immunoassay for PSA detection.

Single-wall carbon nanotubes (SWNT) with [Ru-(bpy)_3_]^2+^-doped silica nanoparticles have been studied as an electrochemiluminescent immunosensor for PSA detection (Figure 9). The doped silica nanoparticle provides significant electrochemiluminescent signal amplification by trapping thousands of [Ru-(bpy)_3_]^2+^ (RuBPY) and can be detected with a much simpler measurement systems than the commercial bead-based electrochemiluminescence (ECL) assays. Thus, an ultrasensitive amperometric immunosensor for PSA detection has been designed based on CNTs [81].

**Figure 10.**
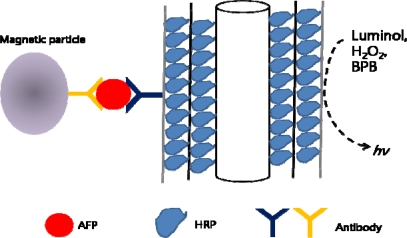
Multilayered enzyme-coated CNTs as labels for chemiluminescence immunoassay.

A multilayered enzyme-coated carbon nanotube design has been studied as an ultrasensitive chemiluminescence immunoassay (CLIA) for detecting AFP in human serum samples. Horse radish peroxide (HRP) was absorbed into MWNTs, allowing maximized ratio of HRP/antibodies for sensitivity enhancement. After separating the MWNT-AFP by applying magnetic beads with antibodies, bromophenol blue (BPB) and H_2_O_2_ was added to the separated solution. The chemiluminescence reaction was triggered by injecting luminol into the solution (Figure 10). This system proved to have a detection limit that was two orders of magnitude lower than standard ELISA method [82].

**Figure 11.**
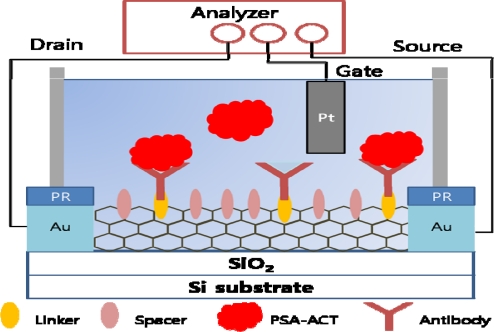
Set up of CNT-FET with a linker and a spacer for the maximized sensitivity.

As mentioned above, by using CNTs label-free detection of biomolecules is possible. As well, due to their advantages many studies have been conducted on the development of CNT based biosensors. CNT field effect transistor (FET) based biosensors are especially of great interest [83]. CNTs form the conducting channel in a transistor configuration and interact with introduced analytes. Analytes interact with CNTs where 1) charge transfer may occur from analyte molecules to the CNTs or 2) the analytes can act as scattering potential across the CNTs. A relationship between the concentration of the analyte and conductance or potential can be monitored and biosensing can be done based on this system. A real-time detection of PSA-ACT complex with CNT-FET has been developed and by providing sufficient space between each antibody on the CNT, the sensitivity of the system was maximized (Figure 11). This spacing is a crucial element of sensing biomarkers in a buffer solution since the target is required to be in a distance within the Debye length to give a large gating effect. Using 1-pyrene butanoic acid succinimidyl ester (PASE) as a linker and 1-pyrenebutanol (PB) as a spacer at a specific linker-to-spacer ratio on SWNTs, conductance of CNT-FET could be largely enhanced [84].

Another interesting study has been done using CNT-FET for biomarker detection in breath samples [85]. A sensor array of FETs based on random networks (RNs) of SWNTs for detection of lung cancer biomarkers from exhaled breath samples was designed, showing a clear discrimination between volatile organic compounds (VOCs) of healthy controls and patients with lung cancer. Conductance response of RN-CNT FETs to nonpolar biomarkers in gas phase was obtained by scattering of carriers and response of same devices to polar biomarkers was achieved from charge transfer from polar adsorbates [86]. Through principal components score plots, a significant difference was observed between healthy controls and cancer patients.

### 3.5. Nanowires

Various nanowires have also been applied to biomarker detection including silicon nanowires [86–89], In_2_O_3_ nanowires [77], gold nanowires [90,91], conducting polymer nanowires [92].

**Figure 12.**
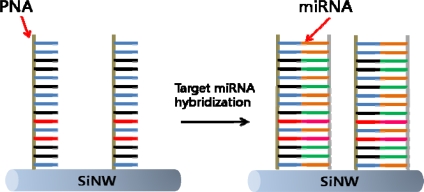
Target miRNA detection via PNA functionalized SiNW.

Silicon nanowires (SiNW) are semiconducting nanowires with exceptional physical, optical, electronic properties, and excellent biocompatibility [93–96]. As well, since silicon is a well studied material, the surface of the nanowires can be modified with well-known methods. This advantage makes itself a promising platform for sensitive detection of biomarkers [97,98]. SiNWs modified with peptide nucleic acids (PNAs) have been used to detect miRNAs that were extracted from Hela cells. This system was developed to detect RNA cancer biomarkers [99]. Resistance change was measured before and after the hybridization of the complimentary PNA and miRNA, and it was shown that this change correlates directly to the concentrations of the target miRNA (Figure 12).

**Figure 13.**
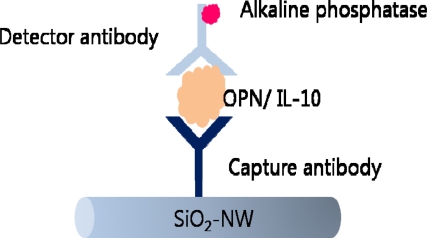
Detection of cancer biomarker through sandwich immunoassay using SiO_2_-NW.

Vascular endothelial growth factor (VEGF), yet another cancer biomarker, has been detected electrically with functionalized SiNWs. This system is also label-free biosensing with both n-type and p-type SiNWs functionalized with anti-VEGF aptamers with current change occurring according to molecular recognition [100]. In addition to silicon nanowires, voltammetric detection of cancer biomarkers using silica nanowires (SiO_2_-NWs) has also been studied. A lung cancer biomarker, interleukin-10 (IL-10) and osteopontin (OPN), has been detected using silica nanowires as templates, through electrochemical alkaline phosphatase (AP) assay (Figure 13).

The streptavidin-coated AP enzyme was attached to the biotinylated detector antibody which then was used to hydrolyze *p*-nitrophenyl phosphate (pNPP) substrate to produce *p*-nitrophenol (pNP). The electro-oxidation of pNP occurrence was measured and the anodic peak current was shown to have a linear relationship with the concentration of the cancer biomarkers [101].

**Figure 14.**
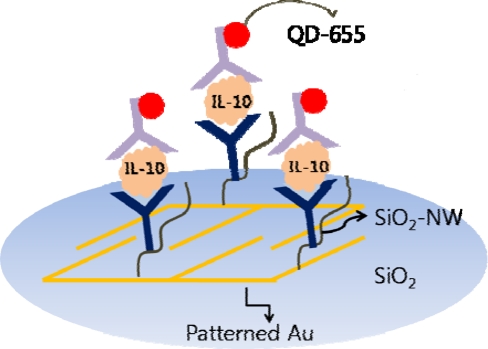
Optical sensing of IL-10 using functionalized SiO_2_-NW and QD labels on a patterned Au substrate.

SiO_2_-NWs with functionalized QDs on a patterned gold substrate have been studied as an optical sensor for cancer biomarkers (Figure 14). The fluorescent intensity was ten-fold than a silica substrate case, and the signal was specified to IL-10 [102]. Metal-decorated SiO_2_-NWs have been studied as an active SERS substrate for cancer biomarker detection [103] with specificity towards IL-10.

**Figure 15.**
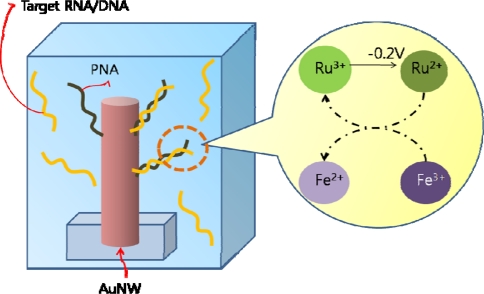
RNA detection via PNA functionalized gold nanowire (AuNW). Solution contains two redox reporter groups; Ru(NH_3_)_6_^3+^ and Fe(CN)6^3–^. Ru(NH_3_)_6_^3+^ is attracted by DNA at electrode surface and through negative potential sweep, Ru(III) is reduced and regenerated by Fe(III) oxidant for multiple turnovers.

Functionalized gold nanowires enhance sensitivity and selectivity for cancer biomarkers detection by providing high surface-to-volume ratio. Electrochemical detection based on alkaline phosphatase enzyme reaction was also used with gold nanowires instead of silicon nanowires [104]. CK-7, a protein expressed in epithelial tissues and can be used as a biomarker to distinguish between different cancer cells, was detected by employing gold nanowires as a template for the enzyme immunoassay. Gold nanowires functionalized with PNA-nanowire have also been investigated as a potential cancer diagnostic system [105]. RNA-DNA hybridization events were detected using an electrocatalytic reporter system, and the cellular and clinical samples did not need any labeling or PCR amplification (Figure 15). As a variation of the nanowire electrodes, a nanostructured microelectrode (NME) system has been developed for mRNA samples of prostate cancer related genes. Palladium NME was constructed on a chip by electrodeposition, and modified with thiol-derivatized PNA probes for mRNA detection. The electrocatalytic signal was monitored for the hybridization events, and the current increase was observed in the presence of the complementary RNA [106].

**Figure 16.**
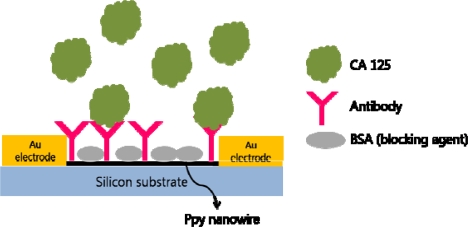
FET system with single Ppy nanowire as semiconducting channel to detect CA 125.

Conducting polymer nanowires were synthesized for chemoresistive sensor device. Polypyrrole (Ppy) nanowires were used in this study and the surface was functionalized with cancer antigen CA 125 antibody (Figure 16). A single Ppy nanowire was integrated into a FET system, just like carbon nanotubes, and the measurements of changes in conductivity due to biomolecule recognition events were measured [107].

### 3.6. Other Nanotechnology (Cantilever and Nanopore)

Microfabricated cantilevers bend according to changes in the environment or changes on their surfaces, and this bending is in the nanometer-scale [108,109]. This nanometer-scale bending allows them to be in the category of nanotechnology even though its whole size is in the micrometer-scale and can be used as a component of biosensors. Microcantilevers have been applied to pH detection [110,111], protein detection [112–117], DNA/RNA-DNA/RNA hybridization events [118–124]. A thorough review on microcantilevers applied in biosensors has been published which discloses that microcantilevers are a unique tool for detecting biomolecules of interest [125].

**Figure 17.**
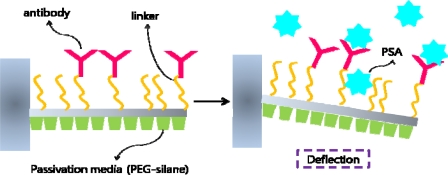
Microcantilever based cancer biomarker detection. Deflection of the cantilever due to antibody-antigen binding is detected by monitoring reflected laser beam.

Recently many developments have been made on microcantilever based biosensors in the area of cancer biomarker detection. Surface stress was measured for antigen-antibody binding, specifically PSA and its antibody [126]. PSA antibodies were conjugated onto the cantilever with specific linkers and in order to reduce background signals, the surface was passivated with PEG-silane. Depending upon the concentration of the antigen, surface stress increased linearly thus providing a system for cancer biomarker detection based on mechanical deflection (Figure 17).

Resonant microcantilevers are also used for sensing biomarkers. Mass change results in a shift in frequency and by observing the shift that originated from the antibody-antigen reaction, an effective biosensor can be designed. A cancer biomarker, CEA, was detected through this method and the microcantilever was coated with a piezoelectric film. In this system, frequency shift occurs by molecular interaction and showed decreasing shift proportional to the target concentration [127]. By fabricating the system with rotating-mode resonant micro-cantilevers, a different type of cancer biomarker, AFP, could be significantly lowered thus providing a potential method of detecting cancer at very early stages [128]. Further development has been made which can be used in undiluted serum sample for cancer detection. Because the resonant frequency was altered by the degree of the molecular interaction, the system could be used as a biosensor for actual human samples [129].

**Figure 18.**
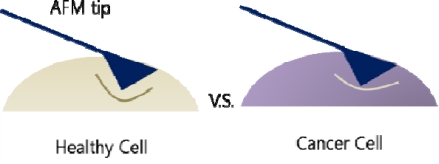
Healthy cell and cancer cell differs in stiffness.

A different approach in detecting cancer cells is measuring the cell stiffness. The cell stiffness here is itself the biomarker thus making it possible to differentiate cancer cells from normal healthy cells. Atomic force microscopy (AFM) is a widely used instrument to measure infinitesimal forces and in this biosensing system, the elasticity of the cells is measured (Figure 18). Histograms of the measured elasticity showed significant difference between cancer cells and normal cells, with cancer cells having a stiffer elasticity and a narrower distribution [130].

**Figure 19.**
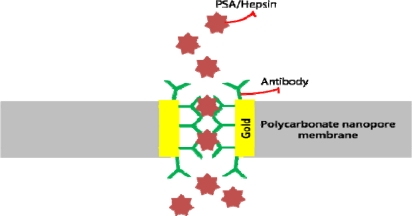
A Nanopore applied in antigen detection. The impedance change is monitored.

Nanopores, including synthetic, artificial, and protein-based, have been of great interest to many researchers for detecting biomolecules. Various studies have been conducted on nanopores for sensing biomolecules including DNA [131–133], proteins [134,135], ions [136,137], and drug molecules [138]. Nanopore membranes functionalized with antibodies was used to detect hepsin and PSA (Figure 19). Electrochemical impedance spectroscopy was used to monitor changes in ionic conductance before and after the antigen-antibody interaction. Results showed increased impedance after the specific interaction, and a linear relationship of change in impedance *versus* antigen concentration was recorded [139].

Methylated DNA can be used as cancer biomarkers for cancer diagnosis and these DNA strands can also be detected by using nanopores. Synthetic nanopores in Si_3_N_4_ membranes have been fabricated and the permeation of methylated DNA passing through the pore was measured. Electric field was used to force translocation of a single DNA across the membrane. The degree of methylation is detected by forcing one molecule to go through the pore at a time and measuring the voltage threshold for each DNA. By being able to discriminate methylation level, it will be possible to apply this system to cancer diagnosis [140].

**Figure 20.**
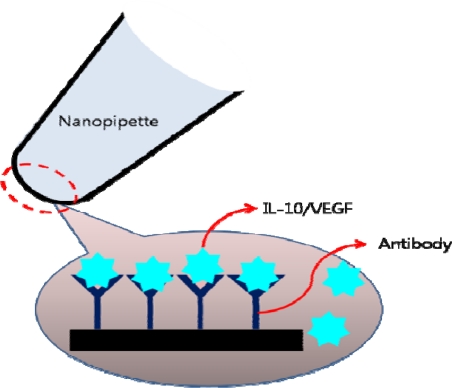
Nanopipette detecting IL-10 or VEGF.

As a type of synthetic nanopores, fabricated nanopipettes have also been studied and developed for sensing applications for DNAs [141–143], and ions [144], and proteins [145]. Currently, methods of label-free-detection of cancer biomarkers have also emerged. Binding of the target molecule on to the surface of the fabricated nanopipette alters the ionic current due to partial blocking or change in the surface charge [146]. This scheme provided a method of detecting VEGF and IL-10, which bound to the surface of the pipette through the capturing antibodies (Figure 20).

## 4. Conclusions

This review summarized recent developments in cancer detection methods with an emphasis on nanotechnology. Nanomaterials have unique features that are attractive, and can be applied to biosensing. The development of various nanomaterials and nanotechnology has enabled detection of cancer biomarkers with great precision and sensitivity that could not be achieved before. The low detection limit obtained by nanotechnology is expected to contribute immensely to the early detection and accurate prognosis of cancers. Since it is of huge importance to be able to diagnose cancer as early as possible, many studies are being conducted on developing sensing mechanisms that will push down the detection limit as far down as possible. As well, various new biomarkers can be discovered and verified with such sensitive tools. It is therefore highly anticipated that in the near future, nanotechnology shall help to detect cancer at an early stage and monitor the disease with much greater precision. It must be however noted that these new technologies must be validated critically before applying them for clinical diagnosis. Although detecting cancers at early stages is very attractive, factors such as probability of getting false positive/negative and impact of nanomaterials on human and environment should be fully understood.

## Figures and Tables

**Table 1.** Current cancer biomarkers in use.

***Cancer***	***Markers***	***Characteristics***	***Typical Sample***
Prostate	PSA (Prostate specific antigen), total and free	High sensitivity in all stages; also elevated from some non-cancer causes	Blood [1,2]
	PSMA (Prostate specific membrane antigen)	Levels tend to increase with age	Blood [3]
Breast	CA 15-3, 27, 29 (Cancer antigen 15-3, 27, 29)	Elevated in benign breast conditions. Either CA 15-3 or CA 27, 29 could be used as marker	Blood [1]
	Estrogen receptors	Overexpressed in hormone-dependent cancer	Tissue [4]
	Progesterone receptors		Tissue [4]
	Her-2/neu	Only 20∼30% of patients are positive to Her-2 oncogene that is present in multiple copies	Tissue [1,5]
Lung (non-small cell)	CEA (Carcinoembryonic antigen)	Used in combination with NSA to increase specificity, used also for colon cancer detection	Blood [6]
Lung (small cell)	NSE (Neuron-specific enolase)	Better sensitivity towards specific types of lung caner	Blood [6]
Bladder	NMP22 (Matritech’s nuclear matrix protein), BTA (Bladder tumor antigen)	NMP-22 assays tend to have greater sensitivity than BTA assays	Urine [1,7]
Pancreatic	BTA	Composed of basement membrane complexes	Urine [1,7]
	CA 19-9 (Carbohydrate antigen 19-9)	Elevated also in inflammatory bowel disease, sometimes used as colorectal cancer biomarker	Blood [1,8,9]
Epithelial ovarian cancer (90 % of all ovarian cancer)	CA 125 (Cancer antigen 125)	High sensitivity in advanced stage; also elevated with endometriosis, some other diseases and benign conditions	Blood [1,10]
Germ cell cancer of ovaries	CA 72-4 (Cancer antigen 72-4)	No evidence that this biomarker is better than CA-125 but may be useful when used in combination	Blood [11]
	AFP (Alpha-fetoprotein)	Also elevated during pregnancy and liver cancer	Blood [1,12]
Multiple myeloma and lymphomas	B2M (Beta-2 microglobulin)	Present in many other conditions, including prostate cancer and renal cell carcinoma.	Blood [13]
	Monoclonal immunoglobulins	Overproduction of an immunoglobulin or antibody, usually detected by protein electrophoresis	Blood, urine [14]
Metastatic melanoma	S100B	Subunit of the S100 protein family	Serum [15]
	TA-90 (Tumor-associated glycoprotein Antigen)	Could be used to monitor patients with high risks of developing the disease	Serum [16]
Thyroid	Thyroglobulin	Principal iodoprotein of the thyroid gland	Serum, tissue [17]
Thyroid medullary carcinoma	Calcitonin	Secreted mainly by parafollicular C cells	Blood, serum [18]
Testicular	hCG (Human chorionic gonadotropin)	May regulate vascular neoformation through vascular endothelial growth factor (VEGF)	Serum [1,19]
Waldenstrom’s macroglobulinemia (WM)	Monoclonal immunoglobulin M	The larger size and increased concentration of the monoclonal protein leads to serum hyperviscosity, the most distinguishing feature of WM	Blood, urine [20]
Lymphomas	B2M	Present in many other conditions, including prostate cancer and renal cell carcinoma	Serum [21]
Lung (non small cell), epithelial, colorectal, head and neck, pancreatic, or breast	EGFR (Her-1)	Binding of the protein to a ligand induces receptor dimerization and tyrosine autophosphorylation and leads to cell proliferation	Tissue [1, 22]
Colorectal, lung, breast, pancreatic, and bladder	CEA (Carcinoembryonic antigen)	Subtle posttranslational modifications might create differences between tumor CEA and normal CEA	Serum [1,23,24]
T-cell acute lymphoblastic leukemia (T-ALL)	PTK7	Membrane-bound surface protein of whole cells, and can be used to detect circulating tumor cells as targets	Blood [25]
